# Exploring the Predictive Value of Circulating Cell-Free DNA Within a Multiparameter Panel for Hepatocellular Carcinoma Detection

**DOI:** 10.3390/life16071079

**Published:** 2026-06-27

**Authors:** Ioana Manea, Speranta Maria Iacob, Razvan Iacob, Alina-Veronica Ghionescu, Andrei Sorop, Roxana Elena Saizu, Daria-Ana-Arina Gheorghe, Delia Prisecariu, Simona Olimpia Dima, Liliana Simona Gheorghe

**Affiliations:** 1 Carol Davila University of Medicine and Pharmacy, 020956 Bucharest, Romania; 2 Center of Excellence in Translational Medicine, Fundeni Clinical Institute, 022328 Bucharest, Romania; 3 Center for Digestive Diseases and Liver Transplant, Fundeni Clinical Institute, 022328 Bucharest, Romania

**Keywords:** hepatocellular carcinoma, circulating cell-free DNA, liquid biopsy, fragmentomics, alpha-fetoprotein, cirrhosis, biomarkers

## Abstract

Background: Hepatocellular carcinoma (HCC) is one of the most common and deadliest cancers worldwide. Alpha-fetoprotein (AFP), a widely used and accessible tumoral marker, has limited performance in the early detection of HCC among high-risk populations. This study aims to evaluate the potential added value of ccfDNA (circulating cell-free DNA) fragment size, alone or in a multiparameter panel, using accessible, feasible ccfDNA analysis. Methods: A prospective cohort of 125 patients with chronic liver disease was analyzed. Patients with incomplete clinical or laboratory data and patients without cirrhosis were excluded from the final analysis. Nonparametric tests, logistic regression and ROC curve analysis were performed. ccfDNA fragment size was measured using on-chip electrophoresis. Results: ccfDNA fragment size was significantly lower in the cirrhosis-HCC subgroup compared to the cirrhosis-only subgroup (*p* < 0.001). While AFP remains an independent predictor of HCC among cirrhosis patients, ccfDNA fragment size did not prove to be an independent predictor in this cohort. AUROC (area under the receiver operating characteristic curve) analysis revealed that a combined model of AFP, age, liver reserve, and ccfDNA fragment size did not perform better than the corresponding panel without ccfDNA. Moreover, after DeLong comparison, the difference between the two AUROCs proved statistically insignificant. Age and platelet count remain the strongest independent predictors in our exploratory cohort. Conclusions: Although ccfDNA fragment size proved to be lower in the HCC subgroup, its statistical significance fades when included into a multimarker panel. However, all panels should undergo further validation in a larger cohort, in order to better assess the individual contribution of each parameter and to discriminate between added diagnostic value and confounding effect of age and liver reserve parameters.

## 1. Introduction

Liver cancer is one of the most common malignancies worldwide. With a reported incidence of 866,136 new cases in 2022, it is the 7th most frequent cancer, as per the World Health Organization database. In terms of mortality, according to Cancer Today data, liver cancer is the 3rd deadliest malignancy, after lung and colorectum cancer, with 758,725 deaths in 2022 [1].

The liver develops from endodermal cells of the ventral foregut, which give rise to hepatocytes and biliary epithelial cells during embryogenesis [2]. Hepatocytes constitute the major functional cell population of the liver and represent the cell of origin for hepatocellular carcinoma (HCC), the most common primary liver malignancy. Early detection of HCC remains paramount for the efforts to reduce disease burden and mortality. Since the vast majority of HCC cases emerge on a cirrhotic liver, early diagnosis remains challenging due to the underlying condition, despite the ongoing advances in diagnostic imaging and surveillance strategies.

Alpha-fetoprotein (AFP) is a 70 kDa carrier protein, predominantly synthesized by the developing liver. It is highly expressed during fetal development and is associated with the growth and differentiation of several tissues. AFP may exert pro-oncogenic and anti-apoptotic effects in adult cells, promoting cellular proliferation, motility and invasiveness in HCC cell lines. Moreover, inhibition of AFP-related signaling pathways has been shown to induce apoptosis in malignant cells, further supporting its potential biological role in tumor progression [3]. Serum AFP may be elevated by several conditions as follows: acute or chronic hepatitis, cirrhosis, HCC, intrahepatic cholangiocarcinoma, gastric cancer, colitis, germinal cell tumors, and at the onset of pregnancy [4]. Several elements may impact AFP accuracy in discriminating between HCC and non-HCC patients with chronic liver disease as follows: etiology of the liver disease, ethnicity, HCC prevalence in the studied population, and the tumor burden of HCC [5,6]. Viral hepatitis remains one of the major drivers of HCC development. Beyond the presence of chronic HBV infection itself, specific viral mutation profiles have also been associated with increased hepatocarcinogenic potential, highlighting the biological heterogeneity of virally induced HCC [7].

According to AASLD most recent guidelines, HCC surveillance for cirrhotic patients should be performed every 6 months, using ultrasound (US) “with or without AFP”. If tested, the AFP cutoff is considered 20 ng/mL [8]. However, the sensitivity of US in HCC surveillance varies significantly between studies, from 45% to 63% in early-stage HCC, performing better only in later stages (sensitivity over 90%) [9,10,11,12,13].

Circulating cell-free DNA (ccfDNA) consists of small DNA fragments, usually approximately 200 base pairs (bp) in length—essentially, the amount of DNA contained by a nucleosome; it contains both tumoral (ctDNA) and nontumoral DNA [14]. Multiple studies evaluating liquid biopsy and its potential role in HCC diagnosis, particularly through the analysis of ccfDNA, have been published in recent years. In a review by Ng et al., the authors evaluated the diagnostic and prognostic applications of ccfDNA concentration, methylation patterns and tumor-associated mutations in HCC, highlighting several studies in which ccfDNA-based biomarkers achieved AUROC values ranging from approximately 0.70 to above 0.90 depending on the analytical approach [15]. Zhang et al. presented liquid biopsy as a noninvasive strategy capable of reflecting the molecular and genetic characteristics of HCC through peripheral blood analysis, noting that several ccfDNA methylation panels demonstrated diagnostic performances exceeding AUROC values of 0.80–0.90, particularly for early-stage HCC detection [16]. Similarly, Wu et al. focused on the utility of ctDNA fragmentation profiles, methylation abnormalities and somatic mutations for early diagnosis and therapeutic monitoring. Several ctDNA-based assays had AUROC values frequently exceeding 0.80 [17]. Finally, Chen et al. conducted a large-scale multicenter study in which ccfDNA analysis revealed potential clinical applicability as a complementary surveillance tool in high-risk populations, for the detection of early/very early HCC (Barcelona stage A/0) [18]. Low-coverage whole-genome sequencing (WGS) has been used to establish fragmentation patterns of ccfDNA for multiple malignancies (including HCC), while deep-coverage WGS is usually employed for mutation profiling.

The present study was designed as an exploratory, hypothesis-generating analysis rather than a comprehensive fragmentomic profiling effort comparable to sequencing-based approaches. It aims to assess whether a simplified, low-cost measurement of ccfDNA fragment size can provide complementary discriminatory information for HCC surveillance in cirrhotic patients, when integrated with readily available clinical and paraclinical data in middle- to low-income hospital settings.

## 2. Materials and Methods

### 2.1. Study Population

A total of 125 patients were included upon presentation to the Gastroenterology unit at Fundeni Clinical Institute, Bucharest. Each patient was given the Ethics Committee-approved consent form, before inclusion (project approval 5475/02.02.2024). Inclusion criteria for HCC and non-HCC patients are listed in Table 1. Four subjects were excluded from the analysis because they did not fulfill the criteria for cirrhosis.

Due to insufficient clinical or laboratory data by the time of data processing, 22 subjects were excluded, the final cohort containing 99 subjects. The patients were divided into HCC and non-HCC subgroups based on clinical and imaging criteria.

### 2.2. Data Collection

Clinical data were collected from the hospital database as follows: age, sex, diagnosis, etiology of underlying liver disease, Child–Pugh classification, BCLC classification, hepatitis viral infection (both present or previous), type of virus and cirrhosis status (present or absent at the time of sample collection). Only patients with cirrhosis were included in the present analysis.

Laboratory data collected from the hospital database included: AFP, transaminases (ALT, AST), total bilirubin (TBIL), alkaline phosphatase (ALP), gamma-glutamyl transferase (GGT), albumin (ALB), INR (international normalized ratio), creatinine (CREA), urea, and platelets (PLT).

**Figure 1:**
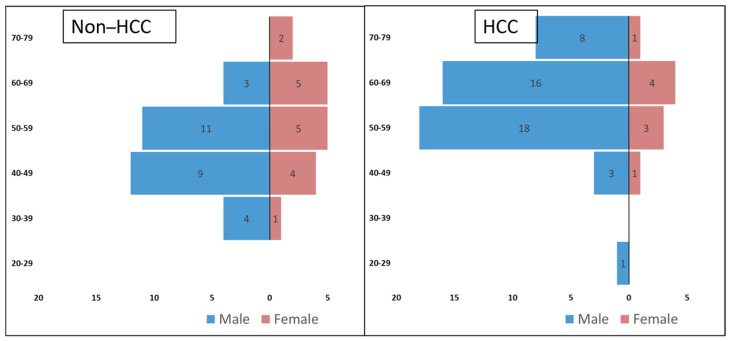
Age and Sex Distribution of Patients, with and without Hepatocellular Carcinoma.

The final structure of the cohort is presented in Figure 1.

### 2.3. Sample Collection and Processing

For each subject included, we collected up to 33 mL of blood, using 3 EDTA (ethylenediaminetetraacetic acid) and 1 clot-activator vacutainers for biobanking and further analysis. All samples were pre-processed within 2 h of collection. Clot-activator vacutainers were centrifuged at 4000 RPM (rotations per minute), and serum was stored at −80 degrees Celsius. EDTA vacutainers were centrifuged at 2000 RPM; plasma and buffy coat were collected. Buffy coat was stored at −80 degrees Celsius, and plasma was centrifuged a second time, at 14,000 *g*. Supernatant was collected and stored in up to 3 aliquots at −80 degrees Celsius until ccfDNA isolation.

Isolation of ccfDNA was performed using QIAamp^®^ MinElute^®^ ccfDNA Midi Kit (Qiagen, Hilden, Germany), using a previously adapted isolation protocol [19].

Quantification of ccfDNA was performed using fluorimetry (Qubit™ dsDNA High Sensitivity Assay Kit, Invitrogen, Thermo Fisher Scientific, Waltham, MA, USA) and fragment size was assessed using on-chip electrophoresis (DNA 1000 Kit or High Sensitivity DNA Kit for 2100 Bioanalyzer Systems and DNA High Sensitivity Kit for 2100 Bioanalyzer Systems, Agilent Technologies, Santa Clara, CA, USA). All electrophoresis chips had valid ladder migration and internal controls (lower and upper markers). Fragment size was automatically measured by the analyzer software for all peaks (also automatically detected).

### 2.4. Statistical Analysis

Data analysis was performed using Microsoft Excel (Microsoft Corp, Redmond, WA, USA), IBM SPSS Statistics for Windows, Version 27.0 (IBM Corp, Armonk, NY, USA) and R version 4.6.0 (R Foundation for Statistical Computing, Vienna, Austria) and RStudio version 2026.04.0 + 526 (Posit Software, PBC, Boston, MA, USA). DeLong comparisons were performed using the pROC package.

Continuous variables were assessed for normality and analyzed using nonparametric tests due to skewed distributions. Mann–Whitney U tests were used for group comparisons. Potential confounding effects of liver functional reserve and viral etiology were explored through subgroup analyses.

Logistic regression analysis was performed to identify independent predictors of HCC and assess the potential complementary value of each parameter. Receiver operating characteristic (ROC) curve analysis was used to evaluate discriminative performance between the HCC and non-HCC subgroups. A combined model was constructed using predicted probabilities derived from logistic regression.

## 3. Results

### 3.1. Cohort Characteristics

Baseline characteristics of the cohort are presented in Table 2 and Table 3, stratified by diagnosis. All patients included were diagnosed with liver cirrhosis, 44 presented no signs of hepatocellular carcinoma at US screening (performed on the day of sample collection, according to current AASLD screening guidelines) and 55 were confirmed with hepatocellular carcinoma via CT scan. The mean age for the cirrhosis subgroup was 51.8 years, with a standard deviation (SD) of 10.8; the mean age of the HCC subgroup was slightly higher—60.3 years—with an SD of 10. AFP median was higher in the HCC subgroup, and had higher heterogeneity compared to the cirrhosis group (the interquartile range—IQR—was wider in the HCC subgroup).

Early-stage HCC was dominant, with 50.9% of the cases, intermediate stage accounted for 20% of the cases and only 29.1% of HCC were advanced or end-stage (BCLC C+D). This stratification is in slight contrast with the most recent data published for a Romanian multicentric cohort (n = 477), which revealed 41.4% early stage, 19.4% intermediate stage and 38.9% advanced and terminal stage HCCs [20]. In both HCC and non-HCC subgroups, the dominant Child–Pugh stages were A and B, with 87.3% and 77.3% respectively. The etiology of the underlying liver disease was similarly distributed throughout both HCC and non-HCC groups, with viral etiology being dominant, followed by alcoholic liver disease. Then, 5% of non-HCC and 14.5% of HCC had mixed alcoholic and viral etiology. Only 9.1% of non-HCC and 12.7% of HCC patients had other etiologies, such as MASLD, cholestatic or cryptogenic.

### 3.2. ccfDNA Measurement Stratified by Underlying Disease

The concentration in the non-HCC subgroup ranged from 1.45 to 105.6 ng/mL of plasma, with a median of 18.8 ng/mL. The concentration in the HCC samples ranged from 1.6 to 209.68 ng/mL, with a slightly lower median of 17.2 ng/mL, suggesting greater heterogeneity within the HCC subgroup. ccfDNA fragment size was assessed using on-chip electrophoresis, with a lower average in the HCC patients, but higher heterogeneity (a 164.9 bp average with a 17.3 SD) compared to the non-HCC patients (a 169.7 bp average with a 6.4 SD).

Liver functional reserve stratification was performed according to the Child–Pugh classification to determine whether the discriminatory performance of ccfDNA concentration between HCC and non-HCC groups was influenced by liver functional status. No statistically significant differences were identified within any Child–Pugh class (all *p* > 0.05).

Stratified analyses according to viral etiology demonstrated no statistically significant differences in ccfDNA concentration between HCC and non-HCC patients within either viral or non-viral subgroups (all *p* > 0.05).

In multivariable logistic regression analysis adjusted for Child–Pugh class and viral etiology, ccfDNA concentration was not independently associated with HCC diagnosis, although a borderline trend was observed (OR 1.019, 95% CI 0.998–1.040; *p* = 0.075). Given these findings, ccfDNA concentration was excluded from further multiparametric analysis for this exploratory study.

Stratified analyses according to Child–Pugh class revealed shorter ccfDNA fragment sizes in HCC patients within Child–Pugh A and B subgroups (Child–Pugh A, *p* = 0.013; Child–Pugh B, *p* = 0.003), whereas no significant difference was retained in Child–Pugh C patients (*p* = 0.358). The persistence of the observed fragment size differences in Child–Pugh A/B patients could be relevant, as these individuals are more likely to be candidates for curative treatment according to the BCLC staging algorithm. In this context, ccfDNA fragment size may warrant further evaluation in larger cohorts. However, the lack of statistical significance for the Child–Pugh C subgroup may be a consequence of the reduced number of patients in this category, and the result should not be considered definitive.

Stratified analyses according to viral etiology also demonstrated shorter ccfDNA fragment sizes in HCC patients compared with non-HCC patients in both viral and non-viral subgroups (viral, *p* = 0.013; no viral infection, *p* = 0.006).

In multivariable logistic regression analysis adjusted for Child–Pugh class and viral etiology, fragment size remained borderline independently associated with HCC diagnosis (OR 0.914, 95% CI 0.835–1.001; *p* = 0.052).

However, since advancing age is associated with an increased risk of malignancy development, age was also considered a potential confounding variable. After adding age as a confounder, fragment size remained borderline associated (OR 0.926; CI 0.850–1.008; *p* = 0.077), while age itself emerged as a relevant parameter in the dataset (OR 1.090; *p* < 0.001). Given the observed association between age and HCC status, additional analyses were performed to explore the relationship between age and ccfDNA fragment size. Correlation analyses revealed a nonsignificant Pearson correlation (r = −0.109, *p* = 0.287) and a weak but statistically significant Spearman correlation (ρ = −0.212, *p* = 0.036). Linear regression analysis identified no significant linear association between age and fragment size (β = −0.101 bp/year, *p* = 0.419; R^2^ = 0.007). In a multivariable linear regression model, with ccfDNA fragment size as the dependent and AFP, age and platelets as covariates, age was not independently associated with ccfDNA fragment size (unstandardized β = −0.021, *p* = 0.880). Among the variables included in the model, age exhibited the smallest effect size, while diagnosis exhibited the highest (unstandardized β = −1.425, *p* = 0.192). None of the parameters revealed statistical significance, and the model had no significant linear association with fragment size (*p* = 0.437; R^2^ = 0.039).

To further explore the potential impact of age imbalance, an exploratory sensitivity analysis was performed in an age-restricted cohort (40–60 years), reducing the mean age difference between groups from approximately 8.5 years to 4.4 years. The age-restricted cohort consisted of 57 cases (29 non-HCC and 28 HCC). The Mann–Whitney U test revealed a statistically significant difference between the HCC and non-HCC groups (Z = −2.926, *p* = 0.003).

### 3.3. Statistical Correlations Between ccfDNA Characteristics, AFP Values and Diagnosis

AUROC (area under the receiver operating characteristic curve) was performed in order to explore the discriminatory performance of an already validated screening marker (AFP), age and compare them to the performance of potentially novel markers.

AFP alone demonstrated moderate diagnostic performance, with an AUROC of 0.736 (95% CI 0.639–0.834). The optimal AFP threshold identified in the present cohort using Youden index analysis was approximately 11.4 ng/mL, lower than the classical diagnostic thresholds proposed in the literature, but higher than the laboratory reference interval (0–9.4 ng/mL).

Age emerged as a strong independent predictor of HCC in multivariable logistic regression analysis. However, when evaluated individually through ROC analysis, age demonstrated only moderate discriminatory performance, with an AUROC of 0.732. This finding suggests that, although advancing age is significantly associated with HCC development, age alone lacks sufficient specificity to function as an isolated diagnostic marker within cirrhotic populations. We further calculated the AUROC for age alone in the exploratory age-restricted (40–60 years) cohort. The performance was lower, 0.696 (95% CI 0.560–0.832), suggesting that age imbalance may partially influence its effect.

ROC analysis of ccfDNA fragment size alone demonstrated moderate discriminatory performance for HCC detection. Since shorter fragment sizes were associated with HCC presence, an inverted variable was used for ROC analysis in order to preserve the conventional interpretation whereby higher test values indicate greater probability of disease. Using this approach, ccfDNA fragment size achieved a rather modest AUROC of 0.716 (95% CI 0.614–0.819), with a maximal Youden index of 0.432.

### 3.4. Performance of Different Combined Models Compared to AFP, Age and ccfDNA Alone

In the present cohort, multiparametric models demonstrated superior performance compared to isolated biomarkers. Since age emerged as a significant independent predictor in multivariable regression analysis despite only moderate isolated discriminatory capacity, age was incorporated into subsequent models alongside the classical marker—AFP. The combined AFP + age model demonstrated a higher discriminatory performance, achieving an AUROC of 0.837 (95% CI 0.760–0.914), supporting the concept that demographic parameters may provide complementary diagnostic information when integrated with serum biomarkers.

Subsequent incorporation of platelet count into the AFP + age model was associated with numerically higher performance, resulting in an AUROC of 0.854 (95% CI 0.780–0.927). Similarly, incorporation of ccfDNA fragment size into the AFP + age model resulted in an AUROC of 0.844 (95% CI 0.768–0.919).

**Figure 2:**
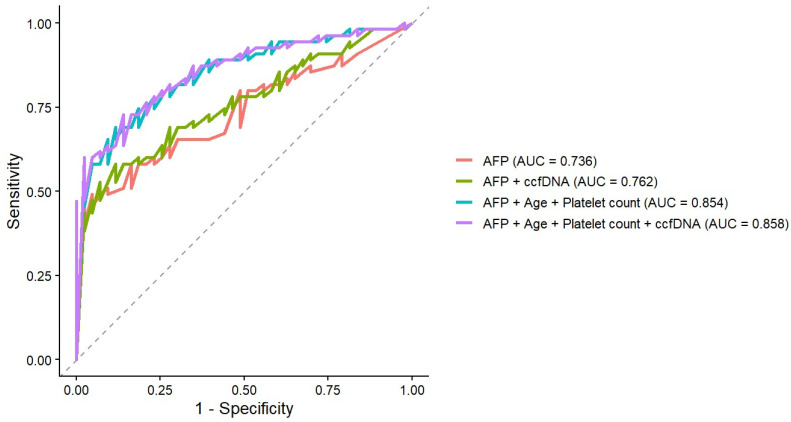
ROC curves for the multimarker panels.

The highest AUROC in the present cohort was achieved by the full multimarker panel integrating AFP, age, platelet count and ccfDNA fragment size. This combined model achieved an AUROC of 0.858 (95% CI 0.785–0.931), with an optimal predicted probability cutoff of 0.588. However, DeLong’s test demonstrated that this improvement was not statistically significant (Z = −0.596, *p* = 0.551). The estimated difference in AUROC was 0.004 (95% CI: −0.018 to 0.010), indicating that inclusion of ccfDNA fragment size did not meaningfully improve discriminatory performance in this cohort, despite its initial moderate predictive value as a single marker. The detailed data are presented in Table 4 and Table 5 and Figure 2.

We also performed bootstrap for the following two best performing panels: AFP and age-maintained stability in both panels; platelet count was borderline stable, while ccfDNA fragment size lost its stability (confidence interval crosses zero). The results are presented in Table 6.

Furthermore, we tested the model with the highest AUROC (AFP + Age + fragment size + platelet count) in the age-restricted cohort. The resulting AUROC was 0.881 (95% CI 0.792–0.969). Bootstrap analysis largely retained the same significance, with age, AFP and platelets remaining stable, and ccfDNA fragment size being unstable. The results are presented in Table 7.

## 4. Discussion

The present study evaluated the potential discriminatory value of circulating cell-free DNA characteristics in cirrhotic patients with and without hepatocellular carcinoma. The main finding of the study is that ccfDNA fragment size was significantly lower in the HCC subgroup compared to cirrhotic controls without HCC. Although fragment size alone demonstrated only moderate discriminatory performance, incorporation into multimarker diagnostic models did not significantly improve overall predictive value, particularly when combined with AFP, age and platelet count.

Interestingly, inclusion of age in multivariable logistic regression analysis attenuated the association between shorter fragment size and HCC diagnosis, shifting the statistical significance from borderline (*p* = 0.052) to nonsignificant (*p* = 0.077). This observation further highlights the potential relevance of demographic parameters in ccfDNA-based analyses. These findings are consistent with the existing literature, as several studies have either correlated ccfDNA fragmentation dynamics with age or proposed ccfDNA as a biomarker of aging. Moreover, this relationship is biologically plausible, since aging influences DNA turnover, apoptosis, chronic inflammation and oncogenesis risk [21,22,23]. In this cohort, correlation analyses revealed only a weak association between fragment size (as the dependent) and age. Both linear regressions performed in the cohort exhibited no statistically significant linear association between fragment size and age, either alone or alongside AFP, platelets and diagnosis. In addition, the full model performance in the age-restricted cohort retained largely the same significance as in the full cohort, despite the decrease in the performance of age alone. However, while creating an age-restricted cohort substantially improved age comparability between groups, it reduced the sample size to 57 participants, thereby limiting statistical power and increasing the risk of model instability and overfitting. Consequently, these analyses should be interpreted as exploratory and hypothesis-generating rather than confirmatory. Together, these observations suggest that the relationship between age and fragment size may not fully account for the observed differences in fragment size between the HCC and non-HCC groups. Nonetheless, they do raise the question of the extent to which age should be regarded as a confounding variable or as a valuable complementary component of multiparametric HCC detection models. Further evaluation in larger, better age-matched validation cohorts is therefore warranted.

Several studies have explored the role of liquid biopsy in HCC diagnosis, particularly through the analysis of circulating cell-free DNA concentration, methylation signatures, and tumor-associated mutations. However, comparatively fewer studies have specifically investigated ccfDNA fragmentation dynamics, most often deriving fragmentomic data from more complex genomic approaches such as low-pass whole genome sequencing, massive parallel sequencing, or ccfDNA size selection followed by whole genome sequencing [24,25,26,27,28,29,30]. Recent experimental models have further emphasized the complexity of molecular pathways involved in hepatic disease development and progression, highlighting the value of biologically informed systems for investigating mechanisms relevant to hepatocarcinogenesis [31]. Although these techniques provide highly valuable information—not only regarding fragment size, but also broader fragmentomic patterns, mutational burden and mechanisms involved in hepatocarcinogenesis—their high cost and the need for specialized laboratory and bioinformatics expertise limit their feasibility for routine exploration in most clinical settings.

In contrast, on-chip electrophoresis represents a considerably simpler and more accessible methodology for fragment size assessment. The technique requires substantially less specialized training, both in terms of laboratory workflow and downstream interpretation, compared to advanced sequencing-based fragmentomic platforms [32,33,34]. Moreover, interpretation of electrophoretic peak profiles is relatively straightforward and does not rely on complex computational pipelines, dedicated bioinformatics infrastructure, or highly specialized personnel. These characteristics facilitate integration into routine clinical laboratory settings, particularly in resource-limited environments or centers without advanced molecular diagnostic infrastructure. The central concept of this exploratory, hypothesis-generating study was therefore to evaluate whether a simpler, lower-cost, and clinically accessible analytical approach could provide incremental value for HCC detection when integrated with information already routinely available in everyday clinical practice.

It is of paramount importance to stress that electrophoretic fragment size does not provide the valuable complex information provided by genomic-based fragmentomics; it cannot replace its diagnostic value and the scope of this analysis was to explore a cheaper, less technically complex assay that may provide incremental value for at-risk population screening, in lower income settings where integration of complex genomic data into clinical practice would be difficult.

Biologically, shorter ccfDNA fragments in HCC patients are plausible and may reflect altered apoptotic and necrotic mechanisms associated with malignant transformation. Tumor-derived DNA fragmentation patterns are believed to differ from nonmalignant ccfDNA due to altered chromatin organization, nuclease activity and nucleosomal packaging. Moreover, chronic inflammation, increased cellular turnover and carcinogenesis-related genomic instability may additionally influence ccfDNA fragmentation profiles in cirrhotic patients with HCC. The biological mechanisms underlying the shorter ccfDNA fragments observed in HCC patients remain incompletely understood. HCC development is driven by complex interactions among genomic, epigenomic, transcriptomic and microenvironmental factors, and recent multi-omic studies have highlighted the value of integrating multiple molecular layers to better characterize disease-specific pathways [35].

In the present cohort, ccfDNA fragment size alone achieved a moderate AUROC of 0.716 after inversion to account for the inverse relationship between fragment size and HCC presence. While this performance is insufficient to support isolated clinical implementation, it suggests that fragment size may capture biological information distinct from traditional biomarkers. This hypothesis was further explored.

AFP remains the most widely used serum biomarker for HCC surveillance and diagnosis despite several recognized limitations. Elevated AFP levels may occur in all types of chronic liver disease, while a substantial proportion of early-stage HCCs may develop without significant AFP elevation. In a large systematic review and meta-analysis, Zhang et al. concluded that an AFP threshold of 200 ng/mL achieved the best overall performance for HCC diagnosis, while lower thresholds (20–100 ng/mL) provided greater sensitivity for early detection [4].

In our cohort, AFP alone demonstrated moderate diagnostic performance, with an AUROC of 0.736 and an optimal cutoff substantially lower than the traditionally cited diagnostic thresholds of 20–200 ng/mL. Importantly, these findings should not be interpreted as a proposal for redefining universal AFP cutoff values for HCC diagnosis, but rather as a cohort-specific observation generated within an exploratory surveillance context.

An interesting finding of the present study was the higher platelet count observed in the HCC subgroup. This observation should be interpreted cautiously, as platelet count is strongly influenced by the severity of portal hypertension and underlying liver dysfunction. Despite the balance in Child–Pugh class distribution, higher platelet counts observed among HCC patients may reflect differences in cirrhosis severity and liver reserve rather than a direct HCC-associated biological signal. Consequently, platelet count may act, at least in part, as a surrogate marker of preserved hepatic function, and residual confounding by cirrhosis severity cannot be completely excluded.

The highest discriminatory performance in this cohort was achieved by the full multimarker model integrating AFP, age, platelet count and ccfDNA fragment size, which demonstrated an AUROC of 0.858. While progressive increases in Youden index values across successive models suggest improved balance between sensitivity and specificity, the performance after incorporation of fragmentation-related parameters did not translate into statistically significant improvement. Nevertheless, the risk of overfitting increases as the number of variables inside the multivariate logistic regression increases. The increase in AUROC proved to be statistically insignificant; thus, ccfDNA fragment size adds no incremental benefit within the multimarker surveillance-oriented approaches for HCC detection in this cohort. The use of multiparametric models in HCC is not limited to surveillance and diagnosis. Similar approaches have been successfully applied in other clinical contexts, including prediction of portal vein tumor thrombosis, further illustrating the potential value of integrating multiple clinical and biological variables into risk stratification frameworks [36]. However, compared to genome-wide fragmentomic approaches, ccfDNA fragment size assessment by on-chip electrophoresis represents a simpler and more accessible methodology, with lower associated costs and reduced requirements for highly specialized laboratory processing and bioinformatic analysis and may warrant further exploration in larger cohorts.

## 5. Limitations

Several limitations of the present study should be acknowledged. Firstly, the study cohort was relatively small and derived from a single center, limiting the generalizability of the findings. The relatively small sample size may increase the risk of overfitting in multivariable models; given the 55 HCC events available, the multivariable analyses should be considered exploratory and hypothesis-generating, because coefficient estimates may be unstable due to the borderline events-per-variable ratio (11 events per variable). Furthermore, internal validation procedures (such as k-fold internal validation) were not performed because the limited sample size would have further reduced the effective training data and yielded unstable estimates. Secondly, the exploratory cross-sectional design does not allow assessment of longitudinal ccfDNA fragmentation dynamics, and it cannot provide confirmation that participants in the control group did not harbor occult HCC or predictive changes preceding HCC diagnosis, at enrollment. Thirdly, the study population consisted exclusively of cirrhotic patients undergoing HCC surveillance, and therefore the findings may not be directly applicable to noncirrhotic HCC populations. Additionally, ccfDNA analysis relied on dominant fragment peak determination rather than high-throughput genome-wide fragmentation profiling or next-generation sequencing-based approaches. Although this simplified methodology may improve accessibility and reproducibility, it will also limit the depth of fragmentation analysis. The method employed by this proof-of-concept study was designed for exploring the incremental value of ccfDNA characteristics, for surveillance of at-risk populations in middle- to low-income hospital settings. Finally, external validation in larger prospectively enrolled independent cohorts incorporating follow-up imaging and clinical surveillance of control participants is necessary before potential clinical value of the proposed multimarker models can be considered.

## 6. Conclusions

ccfDNA fragment size differs significantly between cirrhotic patients with and without HCC. Although fragment size alone demonstrated moderate discriminatory performance, incorporation into combined panels with AFP, age and platelet count (all readily available tests in a hospital setting) did not significantly improve overall discrimination between HCC and cirrhosis-only patients. Larger external validation studies are required to further evaluate the potential utility of this accessible liquid biopsy approach in HCC surveillance as well as the predictive value of multimarker panels comprising AFP, platelet count and other clinical data.

## Figures and Tables

**Table 1:** Patient inclusion criteria.

HCC	Non-HCC
Newly diagnosed HCC	Cirrhosis
No invasive maneuver performed on the liver before blood collection	No invasive maneuver performed on the liver before blood collection
Underlying cirrhosis	No history of other malignancies
No history of other malignancies	Very low probability of HCC during US screening

**Table 2:** Baseline characteristics stratified by diagnosis.

Variable	Non-HCC (n = 44)	HCC (n = 55)
Age (years)	51.8 ± 10.8	60.3 ± 10.0
AFP (ng/mL)	3.8 (2.0–5.6)	9.4 (4.0–43.7)
Platelets (×10^3^/µL)	105.6 ± 48.8	155.3 ± 102.8
ccfDNA concentration (ng/mL)	18.8 (9.9–25.6)	17.2 (11.1–33.9)
ccfDNA fragment size (bp)	169.7 ± 6.4	164.9 ± 17.3

Measurements were expressed as mean ± SD or median (interquartile range).

**Table 3:** Etiology of liver disease and Child–Pugh distribution stratified by diagnosis.

Variable	Non-HCC (n = 44)	HCC (n = 55)
Etiology of liver disease		
ALD	16 (36.4%)	10 (18.2%)
VIR	22 (50%)	30 (54.5%)
ALD + VIR	2 (4.5%)	8 (14.5%)
OTHER	4 (9.1%)	7 (12.7%)
Child–Pugh score		
A	20 (45.5%)	33 (60%)
B	14 (31.8%)	15 (27.3%)
C	10 (22.7%)	7 (12.7%)
BCLC stage of the HCC subgroup		
0	-	7 (12.7%)
A	-	21 (38.2%)
B	-	11 (20%)
C	-	11 (20%)
D	-	5 (9.1%)

ALD = alcoholic liver disease; VIR = viral; “Other” includes: MASLD, cholestatic, cryptogenic.

**Table 4:** Diagnostic performance of multimarker models.

Biomarker/Model	AUROC (95% CI)	Youden Index	Optimal Cutoff
**Fragment size** **(smaller predicts HCC)**	0.716 (0.614–0.819)	0.432	-
**Age**	0.732 (0.631–0.833)	0.422	56.5 years
**AFP**	0.736 (0.639–0.834)	0.444	11.385 ng/mL
**AFP + age**	0.837 (0.760–0.914)	0.523	-
**AFP + age + fragment size**	0.844 (0.768–0.919)	0.570	-
**AFP + age + platelets**	0.854 (0.780–0.927)	0.575	-
**AFP + age + fragment size + platelets**	0.858 (0.785–0.931)	0.588	-

**Table 5:** Sensitivity, specificity, positive predictive value, negative predictive value, and accuracy of single- and multimarker models at the Youden-optimal threshold.

Model	Sn (%)	Sp (%)	PPV (%)	NPV (%)	Accuracy (%)
**AFP**	49.1	95.3	93.1	59.4	69.4
**AFP + Age**	70.9	81.4	83	68.6	75.5
**AFP + Age + Fragment size**	70.9	86	86.7	69.8	77.6
**AFP + Age + Platelets**	69.1	88.4	88.4	69.1	77.6
**AFP + Age + Fragment size + Platelets**	72.7	86	87	71.2	78.6

Sn = sensitivity; Sp = specificity; PPV = positive predictive value; NPV = negative predictive value. Threshold = the corresponding Youden Index.

**Table 6:** Bootstrap analysis for the best performing models.

Model	AFP + Age + Platelets		AFP + Age + Fragment Size + Platelets	
Parameter	*p*	CI	*p*	CI
**AFP**	0.004	0.05; 0.207	0.007	0.043; 0.206
**Age**	0.006	0.03; 0.166	0.008	0.025; 0.166
**Platelets**	0.091	−0.001; 0.19	0.082	0; 0.02
**ccfDNA fragment size**	-	-	0.410	−0.346; 0.21

**Table 7:** Bootstrap analysis for AFP + Age + Fragment size + Platelets, in the age-restricted cohort.

Parameter	*p* (CI)
**AFP**	0.041 (0.011; 0.317)
**Age**	0.009 (0.056; 0.75)
**Platelets**	0.033 (0.002; 0.06)
**ccfDNA fragment size**	0.561 (−0.910; 0.035)

## Data Availability

The raw data supporting the conclusions of this article will be made available by the authors on request.
